# Molecular imaging of angiogenesis after myocardial infarction by ^111^In-DTPA-cNGR and ^99m^Tc-sestamibi dual-isotope myocardial SPECT

**DOI:** 10.1186/s13550-015-0081-7

**Published:** 2015-01-28

**Authors:** Geert Hendrikx, Marijke De Saint-Hubert, Ingrid Dijkgraaf, Matthias Bauwens, Kim Douma, Roel Wierts, Ivo Pooters, Nynke MS Van den Akker, Tilman M Hackeng, Mark J Post, Felix M Mottaghy

**Affiliations:** Department of Nuclear Medicine, Maastricht University Medical Centre (MUMC+), Postbox 5800, 6202 AZ Maastricht, The Netherlands; Department of Biochemistry, Maastricht University, Maastricht, The Netherlands; Department of Physiology, CARIM, Maastricht University, Maastricht, The Netherlands; Cardiovascular Research Institute Maastricht (CARIM), Maastricht University, Maastricht, The Netherlands; Department of Nuclear Medicine, University hospital, RWTH University, Aachen, Germany

**Keywords:** Myocardial infarction, Angiogenesis, CD13, Micro-SPECT

## Abstract

**Background:**

CD13 is selectively upregulated in angiogenic active endothelium and can serve as a target for molecular imaging tracers to non-invasively visualise angiogenesis *in vivo*. Non-invasive determination of CD13 expression can potentially be used to monitor treatment response to pro-angiogenic drugs in ischemic heart disease. CD13 binds peptides and proteins through binding to tripeptide asparagine-glycine-arginine (NGR) amino acid residues.

Previous studies using *in vivo* fluorescence microscopy and magnetic resonance imaging indicated that cNGR tripeptide-based tracers specifically bind to CD13 in angiogenic vasculature at the border zone of the infarcted myocardium.

In this study, the CD13-binding characteristics of an ^111^In-labelled cyclic NGR peptide (cNGR) were determined. To increase sensitivity, we visualised ^111^In-DTPA-cNGR in combination with ^99m^Tc-sestamibi using dual-isotope SPECT to localise CD13 expression in perfusion-deficient regions.

**Methods:**

Myocardial infarction (MI) was induced in Swiss mice by ligation of the left anterior descending coronary artery (LAD). ^111^In-DTPA-cNGR and ^99m^Tc-sestamibi dual-isotope SPECT imaging was performed 7 days post-ligation in MI mice and in control mice. In addition, *ex vivo* SPECT imaging on excised hearts was performed, and biodistribution of ^111^In-DTPA-cNGR was determined using gamma counting. Binding specificity of ^111^In-DTPA-cNGR to angiogenic active endothelium was determined using the Matrigel model.

**Results:**

Labelling yield of ^111^In-DTPA-cNGR was 95% to 98% and did not require further purification. *In vivo*, ^111^In-DTPA-cNGR imaging showed a rapid clearance from non-infarcted tissue and a urinary excretion of 82% of the injected dose (I.D.) 2 h after intravenous injection in the MI mice. Specific binding of ^111^In-DTPA-cNGR was confirmed in the Matrigel model and, moreover, binding was demonstrated in the infarcted myocardium and infarct border zone.

**Conclusions:**

Our newly designed and developed angiogenesis imaging probe ^111^In-DTPA-cNGR allows simultaneous imaging of CD13 expression and perfusion in the infarcted myocardium and the infarct border zone by dual-isotope micro-SPECT imaging.

**Electronic supplementary material:**

The online version of this article (doi:10.1186/s13550-015-0081-7) contains supplementary material, which is available to authorized users.

## Background

The formation of new capillaries from existing micro vessels occurs as a natural healing process following myocardial infarction (MI) [1]. This process, called angiogenesis, is triggered by ischemia and results in partial restoration of blood perfusion in the ischemic zone. Importantly, the extent of angiogenesis is associated with post-infarct remodelling and has positive implications for the prognosis of MI patients [2]. Several therapy approaches, aimed at stimulating angiogenesis in the infarcted area, including gene therapy, intramyocardial administration of pro-angiogenic factors, administration of bone marrow-derived stem cells, and transmyocardial revascularization have been tested [3-12]. To date, results in animal models have been encouraging [3-5]. Nonetheless, the clinical translation of these therapies has proven to be difficult, and the clinical benefit for MI patients has been disappointing and controversial [6-12].

In order to improve treatment options, assessing the efficacy of pro-angiogenic therapies and monitoring the temporal development of angiogenesis, non-invasive methods (e.g. molecular imaging) are warranted. A sensitive and specific molecular imaging marker for angiogenesis, however, is still missing.

Angiogenic vessels demonstrate upregulation of CD13, a membrane-bound aminopeptidase, on activated endothelial cells [13,14]. The cyclic tripeptide Asn-Gly-Arg (cNGR) homes to CD13 expressed on endothelial cells of angiogenic tumour vasculature and angiogenic vessels in the infarcted area and border zones of the myocardium [14-19]. Moreover, evidence was obtained that cNGR does not bind to CD13-positive macrophages in the infarcted myocardium and infarct border zone. This finding was ascribed to cNGR, possibly targeting a subset of post-translationally modified CD13 that might be specific to smaller and perhaps newly formed vessels [17]. Furthermore, using polymerase chain reaction, peak expression of CD13 was shown 7 days after MI in a mouse myocardial infarction model [15]. Non-invasive imaging of CD13 in mice has further been demonstrated with cNGR-labelled paramagnetic quantum dots for molecular magnetic resonance imaging (MRI) [20]. Although angiogenic activity could selectively and non-invasively be depicted in the infarcted heart, molecular imaging with MRI requires high contrast agent dosage, which hampers translation to clinical trials.

The arginine-glycine-aspartic-acid (RGD) motif is known to label angiogenic active endothelium through binding to α_v_β_3_ integrin [21]. In several pre-clinical studies in different neovascularisation models, RGD conjugated agents have been employed [2,22]. However, competition studies in tumour angiogenesis with the NGR and RGD motif demonstrated a threefold higher target homing ratio (tumour/control organ) for NGR than that for RGD [16].

In this study, we designed and developed a CD13 molecular imaging probe that allowed sensitive visualisation using micro-SPECT imaging. In order to target CD13, we used the cNGR tripeptide, having a tenfold higher targeting efficacy than the linear entity [23], to design a non-invasive angiogenesis imaging probe. Thereto, the cNGR tripeptide was conjugated with diethylene triamine pentaacetic acid (DTPA), radiolabeled with ^111^In and evaluated in a mouse model of myocardial infarction in combination with ^99m^Tc-sestamibi in dual-isotope micro-SPECT imaging. In order to determine binding specificity of our newly designed probe, we employed the validated subcutaneous Matrigel plug assay as a model for *in vivo* angiogenesis.

## Methods

### cNGR-DTPA synthesis

NAc-Cys(4MeBzl)-Asn(Xanthyl)-Gly-Arg(Tosyl)-Cys(4MeBzl)-Gly-Gly-Lys(2 chloro-Z)-peptide was synthesised by tert-butyloxycarbonyl (*t*Boc) solid-phase peptide synthesis, on 4-methylbenzhydryl amine resin, as described before [15,24]. After coupling of the last amino acid, the Boc-protecting group was cleaved and the peptide was N^α^ acetylated (10 mL DMF + 230 μL acetic anhydride + 190 μL pyridine, 2 × 2 min). Resin-bound peptide (0.35 g) was cleaved and simultaneously deprotected in anhydrous HF (1 h, 0°C) with 4 vol.% *p*-cresol as a scavenger and the product was precipitated with ice-cold diethyl ether, collected on a glass filter, eluted from the filter using H_2_O/CH_3_CN/TFA (50:50:0.1 *v*/*v*/*v*) and lyophilized to give crude material containing NAc-Cys-Asn-Gly-Arg-Cys-Gly-Gly-Lys-NH_2_. Subsequently, 31.1 mg crude NAc-Cys-Asn-Gly-Arg-Cys-Gly-Gly-Lys-NH_2_ was dissolved in 50 mL 0.1 M TRIS buffer (pH 8), containing 1 M Gn HCl. The resulting solution was stirred for 3 h at room temperature to form the internal disulfide bond. Reaction progress was monitored by analytical HPLC, and after reaction completion, oxidised NAc-Cys-Asn-Gly-Arg-Cys-Gly-Gly-Lys-NH_2_ was purified by semi-preparative HPLC.

Cys-DTPA was coupled to the C-terminal lysine residue of cNGR peptide through a bifunctional succinimidyl ester-maleimide linker, sulfo-SMCC [25]. Therefore, 6.3 mg (7.6 μmol) *cyclic*(NAc-Cys-Asn-Gly-Arg-Cys-Gly-Gly-Lys; cNGR) and 3.5 mg (8.0 μmol; 1.1 eq) sulfo-SMCC were dissolved in 2 mL 0.2 M phosphate buffer (pH 7.5) containing 6 M Gn HCl. After 3.5 h, the reaction mixture was purified by semi-preparative HPLC. Finally, 4.1 mg (4.1 μmol) SMCC-cNGR and 3.5 mg (6.2 μmol; 1.5 eq) Cys-DTPA were dissolved in 400 μL 6 M Gn HCl, 0.2 M phosphate buffer (pH 6.5). The resulting solution was stirred at 37°C, and reaction progress was monitored by analytical HPLC. After reaction completion, the reaction mixture was purified by preparative HPLC, resulting in 4.2 mg DTPA-SMCC-cNGR (yield 63.4%; overall yield 34.3%).

### Animal studies

MI was induced in 10- to 12-week old male Swiss mice by ligation of the left anterior descending coronary artery (LAD) (*n* = 7), as previously described [26]. The angiogenesis imaging protocol was performed 7 days after the LAD ligation. A group of non-operated male Swiss mice (*n* = 5) was used as control. A variable number of animals were available for analysis per experiment. For SPECT scans, seven MI mice and five control mice were used while for biodistribution purposes ≥5 MI mice and ≥4 control mice were used. Furthermore, a group of six 10- to 12-week old male Swiss mice were bilaterally, subcutaneously implanted in the flanks with Matrigel (Table 1). These Matrigels were supplemented with either the pro-angionenic human fibroblast growth factor 2 (hFGF2, final concentration in Matrigel: 0.15 μg/ml) or phosphate-buffered saline (PBS) solution as a control to specifically induce the presence of angiogenic active endothelium. All animals were held under the guidelines of the animal care facility (Maastricht University). All animal experiments were approved by the Committee for Animal Welfare of Maastricht University.

**Table 1:** **Overview of the number of animals that were subjected to different SPECT scans**

**Scan**	**Scan protocol**	**Injected activity (MBq)**	**Number of Swiss mice used**
*In vivo*; MI mice	8 consecutive time frames (15 min each)	30 to 100 MBq ^111^In-DTPA-cNGR co-injected with 100 to 150 MBq ^99m^Tc-sestamibi	7
*In vivo*; control mice	8 consecutive time frames (15 min each)	30 to 100 MBq ^111^In-DTPA-cNGR co-injected with 100 to 150 MBq ^99m^Tc-sestamibi	5
*Ex vivo*; heart slices of MI mice	1 time frame (240 min)	-	Out of 7 MI mice, 4 were used for *ex vivo* scanning purposes
*Ex vivo*; explanted Matrigels	1 time frame (180 min)	90 to 110 MBq ^111^In-DTPA-cNGR	6^a^

^a^Six Swiss mice were implanted bilaterally subcutaneously with Matrigels (hFGF2-supplemented or PBS); hence, six pairs of Matrigels were scanned.

### Imaging probes

#### ^111^In-DTPA-cNGR

Ten micrograms of DTPA-cNGR was dissolved in 0.5 ml of NaAc buffer (0.5 M, pH6) and radiolabeled with ^111^InCl_3_ (400 to 600 MBq, in ± 0.2 ml 0.1 M HCl, Mallinckrodt, Petten, The Netherlands) at room temperature for 30 min. Product purity was checked by HPLC (Shimadzu Corporation, Columbia, MD, USA) using an Aeris WIDEPORE 3.6u XB-C18 column 250 × 4.6 mm (Phenomenex, Utrecht, The Netherlands) eluted with a gradient from 0.1% trifluoroacetic acid (TFA) in water (0 to 3 min) to acetonitrile (ACN) over the course of 20 min at a flow rate of 1 ml/min.

#### ^99m^Tc-sestamibi

^99m^Tc-sestamibi was prepared with freshly eluted pertechnetate from a ^99^Mo/^99m^Tc generator (UltraTechneKow, Mallinckrodt Medical, Petten, The Netherlands). A fraction of the eluate was added to the lyophilized sestamibi-kit (Mallinckrodt Medical, Petten, The Netherlands), and the product was prepared according to manufacturer guidelines. Quality controls were satisfactory for every synthesis (radiochemical purity >98%).

### Micro-SPECT

#### Imaging protocols

Mice were anesthetised with isoflurane (induction 2.5%; maintenance; 1.5%), a catheter was placed in the tail vein and the animals were positioned in the micro-SPECT camera (MiLabs, Utrecht, The Netherlands).

#### SPECT reconstructions

**Figure 1:**
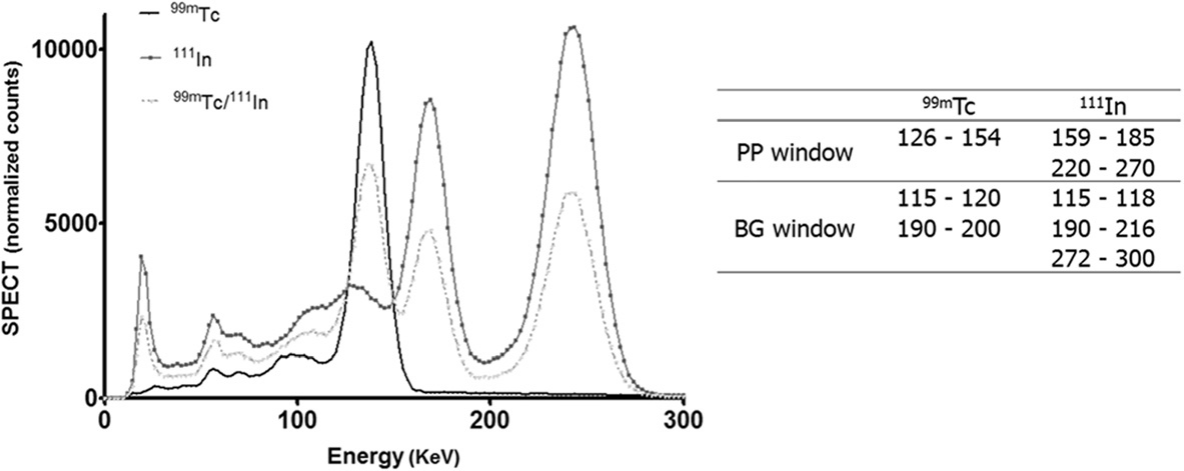
**Energy spectra of** ^**99m**^ **Tc,** ^**111**^ **In and the PP and BG windows used during image reconstruction.**

U-SPECT-II reconstruction software version 2.38 (MiLabs, Utrecht, The Netherlands) was used to reconstruct images [27]. ^99m^Tc and ^111^In images were reconstructed by selecting the photopeak (PP) and background (BG) windows as indicated in Figure 1.

#### SPECT quantification

To allow quantification of radiotracer uptake *in vivo*, conversion factors (CFs) were determined for the 0.6-mm collimator system in a representative phantom (20 ml) for ^99m^Tc and/or ^111^In. During the SPECT reconstruction, the total amount of counts was distributed over the different voxels, resulting in a counts/voxel expression for the average concentration of counts. Taking into account the voxel volume (Vv), the CFs are calculated using equation (*a*):

a $$ \mathrm{Conversion}\ \mathrm{factor}\left(\mathrm{MBq}\right)\kern0.5em =\kern0.5em \frac{\mathrm{Activity}\ \mathrm{concentration}\left(\frac{\mathrm{MBq}}{\mathrm{ml}}\right)}{\frac{\mathrm{SPECT}\ \mathrm{counts}\ \mathrm{per}\ \mathrm{voxel}}{\mathrm{Vv}\left(\mathrm{ml}\right)}} $$

Using this formula, we were able to determine the following CFs: CF_Tc_ = 635 MBq and CF_In_ = 648 MBq.

The PMOD 2.95 cardiac tool PCARD (PMOD Technologies, Zürich, Switzerland) was used to segment the heart in the 17-segment model according to the American Heart Association (AHA) guidelines. For both the ^99m^Tc-sestamibi and ^111^In-DTPA-cNGR images, the uptake was measured for each segment and expressed as mean standardised uptake value (SUV_mean_) using equation (*b*).

b $$ \mathrm{S}\mathrm{U}\mathrm{V} = \left[\mathrm{SPECT}\ \mathrm{counts}\ \mathrm{per}\ \mathrm{voxel} \times \kern0.5em \frac{\mathrm{CF}}{\mathrm{Vv}}\right]\left(\mathrm{MBq}/\mathrm{ml}\right)\kern0.5em \times \kern0.5em \left[\frac{\mathrm{Body}\ \mathrm{weight}\ \left(\mathrm{g}\right)}{\mathrm{Injected}\ \mathrm{dose}\ \left(\mathrm{MBq}\right)}\right] $$

For body weight, we assumed that 1 g of body weight equalled 1 ml.

In order to quantify the absolute uptake of ^111^In-DTPA-cNGR in the different heart pieces, gamma counting was performed using an automated NaI(Tl) gamma counter (Wallac Wizard, Turku, Finland). Additionally, we dissected the vital organs of the mice to study overall biodistribution. The uptake of ^111^In-DTPA-cNGR was determined 2 h post-injection (p.i.) for both MI (*n* ≥ 5) and control animals (*n* ≥ 4). Data were expressed as percentage of injected dose per gram tissue (%ID/g). Urinal excretion was expressed as %ID.

Following *ex vivo* SPECT scans, the explanted Matrigels were kept for gamma counting. Muscular tissue from the hind limb was harvested as a reference. The activity per Matrigel was corrected for ID and weight of the matrigel.

### Statistics

Data were expressed as averages for each group ± standard error of the mean (SEM). To test the significance, we performed unpaired one-tailed student's *t*-test and *p* < 0.05 was considered statistically significant. Data were analysed using Microsoft Excel (version 2010).

## Results

### Chemistry

After cleavage from the solid support with HF, the linear NGR peptide was cyclised by the formation of an internal disulfide bond. This resulted in a mass reduction of 2 by the loss of two protons. MALDI-ToF analysis of the linear peptide showed a mass of 835.47 (calculated: 834.97; C_30_H_54_N_14_O_10_S_2_ [M + H]^+^). For the oxidised peptide, a mass of 833.30 was observed on ESI-MS (calculated: 832.95; C_30_H_52_N_14_O_10_S_2_ [M + H]^+^) Subsequently, a linker that contains both an NHS ester and a maleimide reactive group was coupled to the N^ε^-group of lysine. Finally, the maleimide group was used to couple Cys-DTPA. cNGR-SMCC-DTPA was obtained in an overall yield of 34.3%. ESI-MS of cNGR-SMCC-DTPA: *m/z* C_63_H_102_N_20_O_24_S_3_ [M + H]^+^ calculated monoisotopic mass: 1618.65; found: 1619.72.

### Synthesis of ^111^In-DTPA-cNGR

The labelling yield of ^111^In-DTPA-cNGR was 95% to 99% for every synthesis and did not require further purification.

### Micro-SPECT imaging and analysis

**Figure 2:**
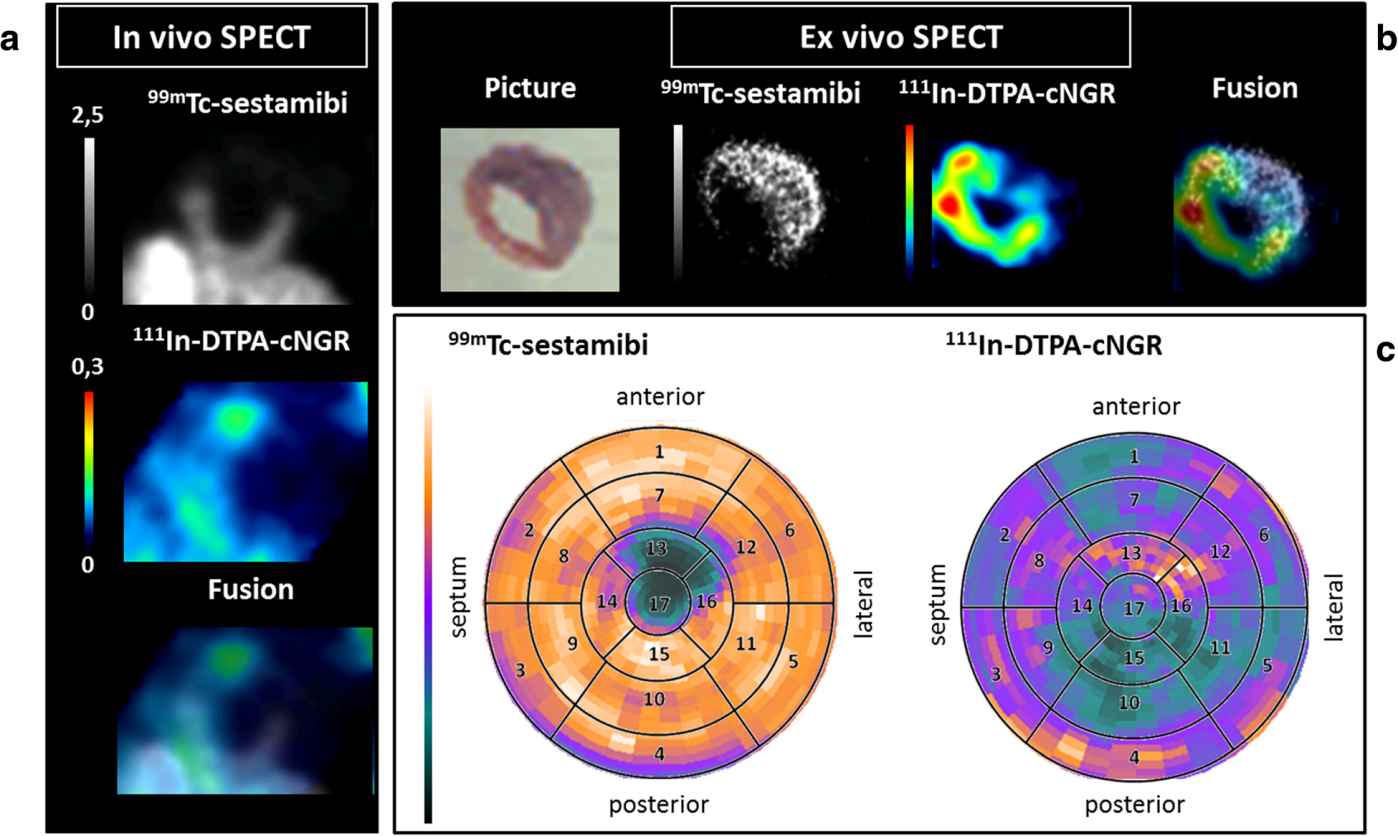
***In vivo*** **and** ***ex vivo*** **transversal tomographic images of an infarcted mouse-heart. (a)** *In vivo* images of ^111^In-DTPA-cNGR uptake, mainly in areas of ^99m^Tc-sestamibi absence. Numerical values on the scale bars indicate the SUV_max_ and SUV_min_. **(b)** *Ex vivo* images of ^111^In-DTPA-cNGR uptake, mainly in areas of ^99m^Tc-sestamibi absence. **(c)** Polar perfusion maps combined with the 17 segment model show that enhanced ^111^In-DTPA-cNGR uptake (orange in areas 13, 16 and 17) occurred mainly in areas with low ^99m^Tc-sestamibi uptake (green in areas 13, 16 and 17), signifying the anterolateral region in the infarcted myocardium.

Transversal tomographic images of the mouse hearts with MI showed decreased uptake of ^99m^Tc-sestamibi in the anterolateral region of the left ventricle 7 days after LAD occlusion (Figure 2), indicating a perfusion defect. The uptake of ^111^In-DTPA-cNGR mirrored that of ^99m^Tc-sestamibi in that the probe was taken up in areas of low perfusion. These uptake patterns were revealed on images of the *in vivo* scans and even more pronounced on images of the *ex vivo* scans (Figure 2). In non-infarcted hearts, the uptake of ^99m^Tc-sestamibi was equally distributed over the whole myocardium while uptake of ^111^In-DTPA-cNGR was limited (data not shown).

**Figure 3:**
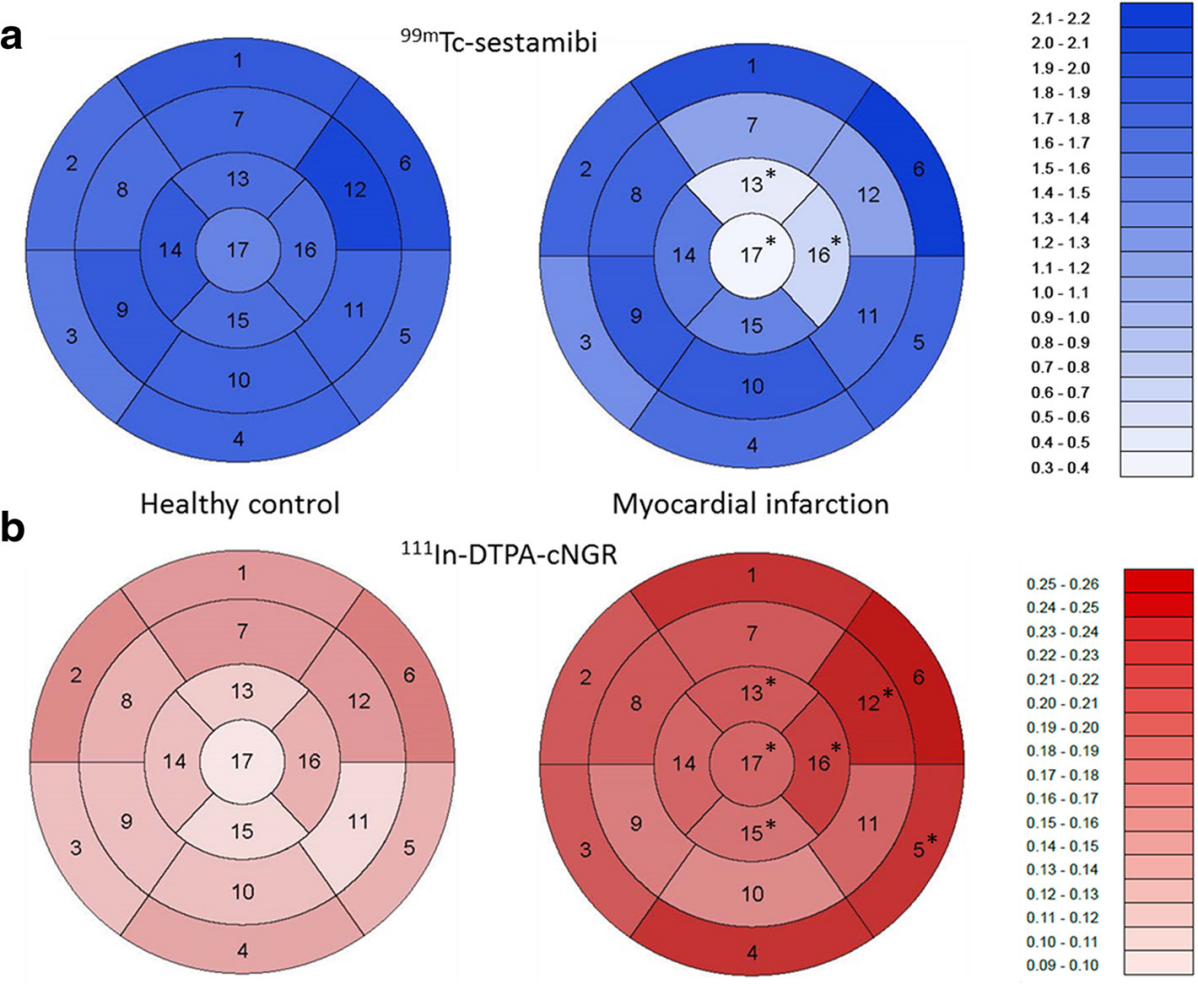
**Overview of cardiac 17 segment models for** ^**99m**^ **Tc-sestamibi and** ^**111**^ **In-DTPA-cNGR SUVs.** In healthy control hearts and MI hearts. **(a)** Cardiac 17 segment models of the healthy control mice and the MI mice indicating colour-coded average ^99m^Tc-sestamibi SUVs per segment. **(b)** Cardiac 17 segment models of the healthy control mice and the MI mice indicating colour-coded average ^111^In-DTPA-cNGR SUVs per segment. Statistical significance was indicated as asterisk (*) (*p* < 0.05).

Standardised uptake values (SUVs) of ^99m^Tc-sestamibi were significantly decreased in the anterolateral region of the left ventricle in infarcted hearts compared to those in the healthy control mice (Table 2 and Figure 3a). In the MI mice, six segments displayed significantly higher ^111^In-DTPA-cNGR uptake compared to that in the healthy control hearts (Table 1 and Figure 3b), of which three had significantly lower ^99m^Tc-sestamibi uptake. The six segments that displayed significantly enhanced ^111^In-DTPA-cNGR uptake were all located in the infarcted myocardium or in the infarct border zone.

**Table 2:** **Overview of the SUVs of** ^**99m**^ **Tc-sestamibi and** ^**111**^ **In-DTPA-cNGR in healthy control mice (** ***n*** **= 5) and MI mice (** ***n*** **= 7)**

	**Standardised uptake values (SUV)**			
	^**99m**^ **Tc-sestamibi**		^**111**^ **In-DTPA-cNGR**	
**Segments**	**Healthy control**	**Myocardial infarction**	**Healthy control**	**Myocardial infarction**
1. Basal anterior	1.87 ± 0.34	1.94 ± 0.36	0.16 ± 0.05	0.24 ± 0.04
2. Basal anteroseptal	1.63 ± 0.28	1.72 ± 0.33	0.16 ± 0.05	0.21 ± 0.03
3. Basal inferoseptal	1.54 ± 0.29	1.38 ± 0.28	0.13 ± 0.06	0.20 ± 0.02
4. Basal inferior	1.77 ± 0.28	1.64 ± 0.24	0.15 ± 0.05	0.23 ± 0.03
5. Basal inferolateral	1.68 ± 0.30	1.78 ± 0.31	0.14 ± 0.04	0.23 ± 0.03*
6. Basal anterolateral	1.95 ± 0.33	2.11 ± 0.39	0.18 ± 0.06	0.26 ± 0.04
7. Mid anterior	1.80 ± 0.30	1.18 ± 0.26	0.15 ± 0.05	0.21 ± 0.02
8. Mid anteroseptal	1.67 ± 0.28	1.73 ± 0.27	0.14 ± 0.06	0.20 ± 0.02
9. Mid inferoseptal	1.80 ± 0.34	1.82 ± 0.30	0.12 ± 0.06	0.18 ± 0.03
10. Mid inferior	1.72 ± 0.28	1.85 ± 0.31	0.12 ± 0.05	0.17 ± 0.03
11. Mid inferolateral	1.71 ± 0.25	1.60 ± 0.32	0.11 ± 0.04	0.20 ± 0.03
12. Mid anterolateral	2.03 ± 0.32	1.15 ± 0.37	0.15 ± 0.05	0.24 ± 0.01*
13. Apical anterior	1.64 ± 0.30	0.49 ± 0.10*	0.11 ± 0.03	0.21 ± 0.02*
14. Apical septal	1.82 ± 0.34	1.55 ± 0.25	0.12 ± 0.04	0.20 ± 0.03
15. Apical inferior	1.67 ± 0.28	1.36 ± 0.35	0.10 ± 0.03	0.18 ± 0.03*
16. Apical lateral	1.60 ± 0.30	0.64 ± 0.24*	0.14 ± 0.04	0.22 ± 0.02*
17. Apex	1.47 ± 0.34	0.39 ± 0.17*	0.10 ± 0.03	0.20 ± 0.03*

Data are displayed as mean ± S.E.M. *p < 0.05, statistical significance.

### Biodistribution of ^111^In-DTPA-cNGR

**Figure 4:**
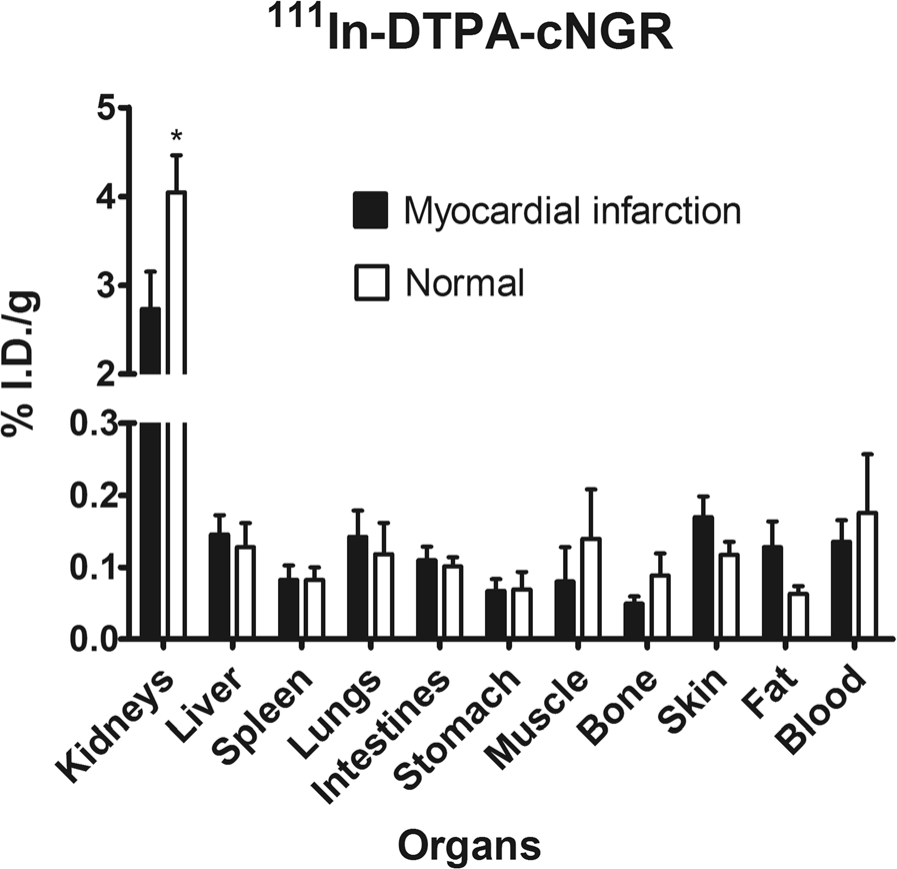
**Comparison of** ^**111**^ **In-DTPA-cNGR uptake in different organs.** Between the MI (*n* ≥ 5) and the healthy control mice (*n* ≥ 4). Significantly higher uptake of ^111^In-DTPA-cNGR was observed in the kidneys of the healthy control mice compared to that in the MI mice (*p* = 0.04). Statistical significance was indicated as asterisk (*) (*p* < 0.05).

Considerable kidney uptake of ^111^In-DTPA-cNGR was observed, which was significantly higher in the healthy control mice than that in the MI mice (Figure 4, 4.04 ± 0.41 vs. 2.73 ± 0.42, *p* = 0.04). The urine (measured as%ID) contained the highest radioactivity. The difference between the healthy control and the MI mice was not significant (92.5 ± 1.17 vs. 82.29 ± 4.87, *p* = 0.08). A specific uptake in other organs was limited and did not reveal any significant differences between the MI mice and the healthy control mice (Figure 4).

**Figure 5:**
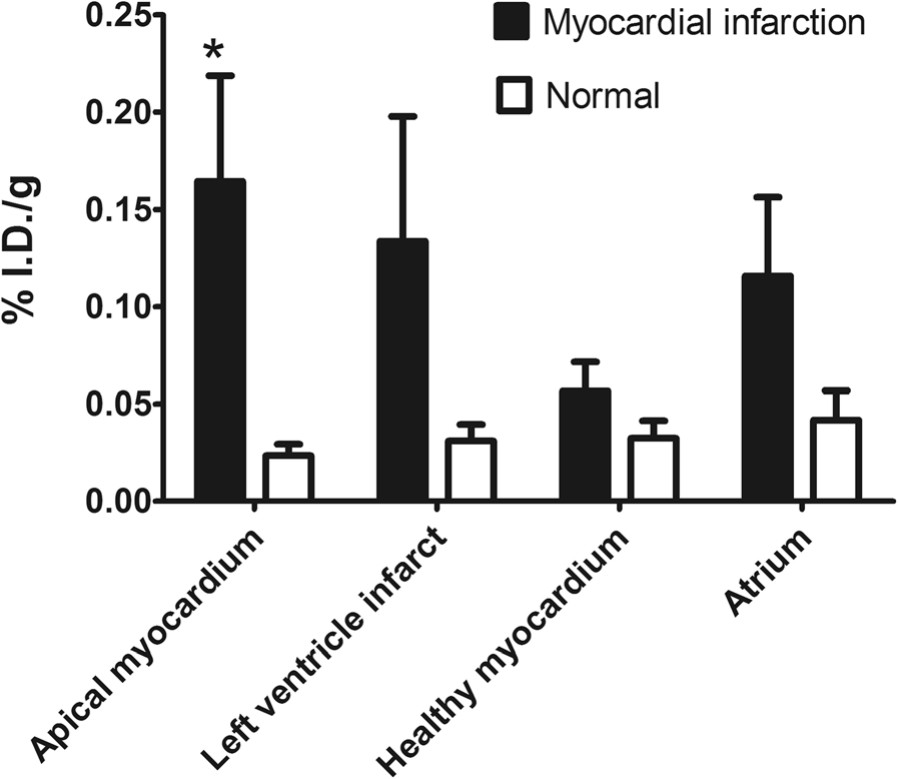
**Uptake of** ^**111**^ **In-DTPA-cNGR.** Uptake of 111In-DTPA-cNGR was significantly higher in the apical region of infarcted hearts compared to that in the healthy control hearts. Uptake of ^111^In-DTPA-cNGR was not significantly different in other regions of the myocardium.

The uptake of ^111^In-DTPA-cNGR was significantly higher in the apical region of the infarcted hearts than in non-infarcted hearts (%ID/g of 0.16 ± 0.0542 vs. 0.02 ± 0.0050, *p* = 0.03). The left ventricular and atrial regions were not significantly different between groups (Figure 5, *p* = 0.09 and *p* = 0.07, respectively). Moreover, uptake of ^111^In-DTPA-cNGR in healthy myocardium of the left ventricle was not significantly different between the MI mice and the healthy control mice (Figure 5).

### Binding of ^111^In-DTPA-cNGR to angiogenic active endothelium

**Figure 6:**
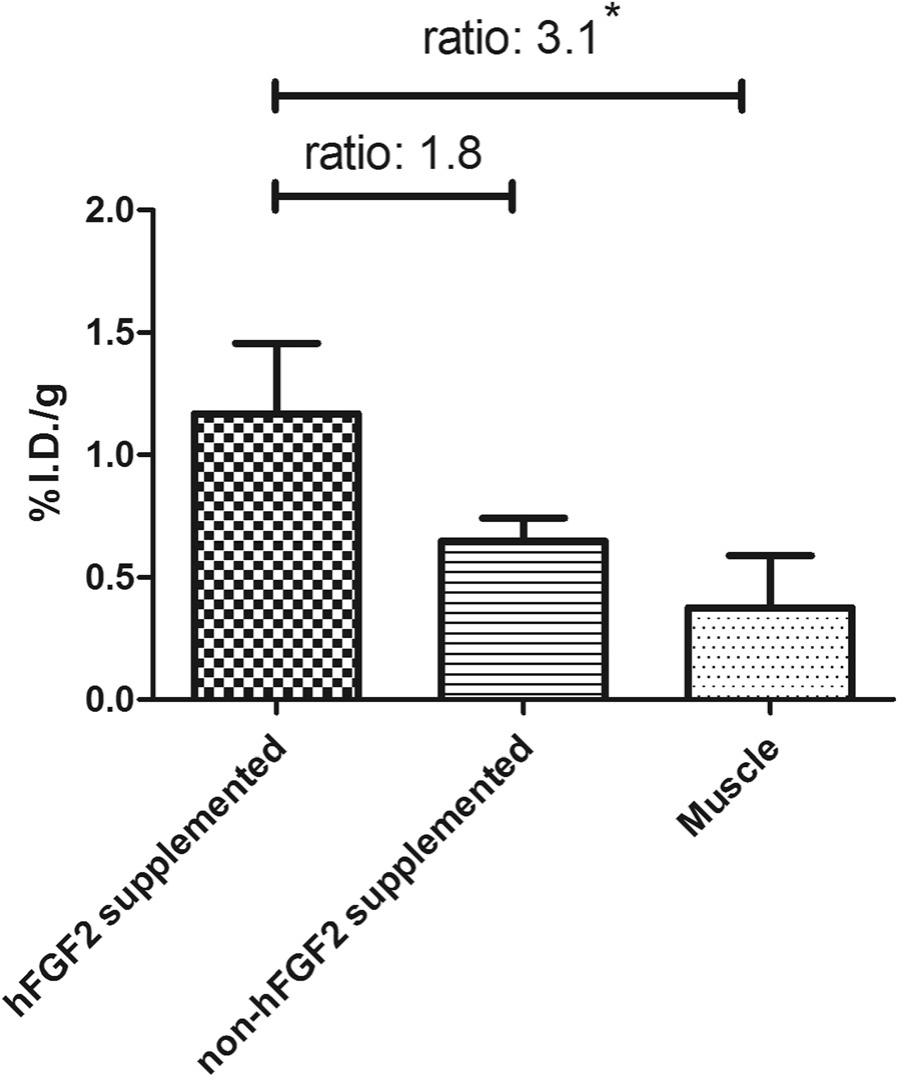
**Uptake of** ^**111**^ **In-DTPA-cNGR was higher in hFGF2-supplemented Matrigels compared with controls.** The uptake of ^111^In-DTPA-cNGR in hFGF2-supplemented Matrigels was significantly higher compared with that in the control muscle tissue while uptake of ^111^In-DTPA-cNGR in the control Matrigels was non-significantly different compared to that in the control muscle tissue. Data are displayed as mean ± S.E.M. Statistical significance was indicated as asterisk (*) (*p* < 0.05).

To visualise specific binding of ^111^In-DTPA-cNGR to angiogenic active endothelium, we bilaterally implanted Matrigel plugs in the flanks of six Swiss mice. On the *in vivo* SPECT images, the hFGF2 supplemented Matrigels were unidentifiable as the kidneys, displaying high uptake, were in too close proximity. Therefore, we explanted each Matrigel and performed *ex vivo* scans. The mean SUV of explanted hFGF2-supplemented Matrigels was higher compared with that in the controls (8.238 × 10^−4^ ± 5.823 × 10^−4^ vs. 2.2235 × 10^−4^ ± 7.001 × 10^−5^, *p* = 0.1527 and ratio 3.7 supplemented vs. non-supplemented) (see Additional file 1: Figure S1). Moreover, gamma counter data of explanted Matrigels indicated a higher uptake of ^111^In-DTPA-cNGR between hFGF2-supplemented and control Matrigels (*p* = 0.0748). Compared with harvested control muscle tissue, a significantly higher uptake of ^111^In-DTPA-cNGR was noticed (*p* = 0.0497) in hFGF2-supplemented Matrigels (Figure 6).

## Discussion

Angiogenesis is associated with post-infarct remodelling and has important implications for prognosis following MI [2]. While previous research has mainly focused on developing pro-angiogenic therapies to stimulate angiogenesis in the infarcted myocardium [28,29], a clinically applicable method for the evaluation of these therapies is still lacking. Most clinical studies thus far have relied on indirect evidence of myocardial angiogenesis. For example, changes in stress-induced ischemia and myocardial perfusion were assessed by treadmill exercise testing and dual-isotope gated SPECT thallium-201/^99m^Tc-sestamibi perfusion SPECT imaging at baseline, day 29, day 57 and day 180 after administration of the pro-angiogenic recombinant-fibroblast growth factor 2 (rFGF2) in a clinical study [9]. In another study, using a single intracoronary infusion of rFGF2, patients experienced increased quality of life (assessed by Seattle Angina Questionnaire) and exercise tolerance (assessed by treadmill exercise testing). Moreover, in this study, MRI demonstrated increased regional wall thickening and a reduction in the extent of the ischemic area compared to baseline [30].

A clinical trial was performed in which patients with exertional angina were treated with the pro-angiogenic recombinant human vascular endothelial growth factor (rhVEGF) [31]. In this trial, no improvement in exercise treadmill test time or myocardial perfusion, as assessed by rest-stress thallium-201/^99m^Tc-sestamibi (gated) SPECT scanning protocol, was detected. However, patients experienced a significant improvement in angina class at day 120 after the first administration of rhVEGF [31]. Furthermore, in a small phase 1 clinical study using VEGF gene transfer, all patients had significant reduction in angina and objective evidence of reduced ischemia was documented using dobutamine ^99m^Tc-sestamibi SPECT imaging. Post-operative left ventricular ejection fraction (LVEF) was either unchanged or improved. Moreover, the Rentrop scoring system, indicating the coronary collateral filling as assessed by coronary angiography, improved in all patients [32].

The pro-angiogenic effect of transmyocardial laser revascularisation (TMLR) was assessed in a small clinical study (seven patients) by Rimoldi et al. While angina scores (Canadian Cardiovascular Society) decreased significantly, exercise tolerance (treadmill testing), 12-min walk distance and the LVEF (assessed by radionuclide ventriculography) remained unchanged. Moreover, despite subjective improvement in some patients, myocardial blood flow (as assessed by ^15^O-labelled water) during dobutamine in lasered segments failed to show any significant increase [33].

Clearly, improved myocardial perfusion or function can be used as indirect evidence for angiogenesis in the infarcted myocardium. Reduced angina scores, increased treadmill exercise times and improved myocardial perfusion as measured by angiography, SPECT, PET and MRI all seem to indicate recovery [34]. However, despite the ability of these methods to indicate recovery, it often takes several months before any improvement can be detected. Therefore, these methods, used in current MI patient care, lack the sensitivity to monitor, or predict, pro-angiogenic treatment. To make direct angiogenesis imaging available for MI patient care, sensitive and specific non-invasive direct angiogenesis imaging probes are required.

To our knowledge, this is the first time CD13 expression was visualised with a nuclear cNGR probe in a mouse model of MI using dual-isotope micro-SPECT imaging. Simultaneous perfusion-imaging enabled us to visualise the angiogenesis process in the border zone of the infarct and the infarcted tissue itself. According to previous studies from our lab, angiogenesis is expected to occur in the infarct area and border zone of the infarct [15,20]. Additionally, in co-localisation experiments using the fluorescently labelled endothelial cell marker anti-CD31-PE, the fluorescently labelled angiogenic active endothelial cell marker anti-CD105-FITC and fluorescently labelled cNGR conjugates, evidence for preferential binding of cNGR conjugates to angiogenic active endothelial cells of capillaries and larger vessels with an inner diameter up to 15 μm was provided [15,20]. Moreover, despite the abundant presence of CD13-positive macrophages, fluorescently labelled cNGR did not bind to anti-CD13-FITC labelled macrophages [15]. In the current study, we used the exact same animal model and the same cNGR moiety, assuring identical experimental conditions. Extrapolating from these data, we therefore consider binding of our ^111^In-DTPA-cNGR imaging probe to be specific for angiogenic endothelium.

Specific binding of our probe is further emphasised by enhanced binding of ^111^In-DTPA-cNGR to angiogenic active endothelium in a specific model for angiogenesis. Unfortunately (hFGF2-supplemented) Matrigels, implanted in the flanks for optimal stimulation of angiogenesis, were unrecognisable on *in vivo* SPECT scans as high renal uptake hampered image analysis. Therefore, we explanted the Matrigels and performed *ex vivo* SPECT scans that indicated higher uptake of ^111^In-DTPA-cNGR in hFGF2-supplemented Matrigels compared with control Matrigels. Moreover, the uptake of ^111^In-DTPA-cNGR in hFGF2-supplemented Matrigels was higher compared with that in the control Matrigels as indicated by gamma counting. hFGF2-supplemented Matrigels displayed significantly higher uptake than control muscle tissue, indicating active angiogenesis.

To use this imaging probe in a clinical setting, the probe should display a high specific target uptake and fast clearance from non-specific organs. The biodistribution characteristics of our probe were studied 2 h post-injection and demonstrated a rapid clearance in the urine which is a favourable clearance pathway. This is in contrast with results for the hydrophilic RGD probe, mainly studied for tumour angiogenesis imaging, that demonstrates unfavourable binding to kidneys, spleen, lungs, liver, and gastrointestinal system thereby hampering the image quality [35].

We simultaneously imaged and quantified cardiac uptake of ^99m^Tc and ^111^In in the AHA 17 segment model. The isotope uptake per segment was quantified using isotope specific CFs, assessed in phantom studies. Furthermore, isotope uptake was expressed in the clinically used SUV parameter, which normalises for injected dose and body weight. Although normalisation for body weight might be considered irrelevant for a rapidly excreted tracer that does not reach a uniform distribution throughout the body, we believe that the body weight bias in our quantification is limited due to the fact that all the animals used in this study were of similar age and body weight.

We found target-specific binding of our new angiogenesis imaging probe as evidenced by the significantly enhanced SUVs in six segments in the infarcted myocardium and the infarct border zone. To even further improve dual-isotope micro-SPECT imaging of perfusion defects and mirroring ^111^In-DTPA-cNGR uptake in the hearts of the MI mice, we are currently pursuing strategies to further enhance probe-target interaction through multivalent binding which may result in higher image quality. The proof of principle of this approach has already been demonstrated in studies from Li et al. and Dijkgraaf et al., in which tumour uptake increased with increasing RGD valency [35,36].

Despite displaying enhanced, specific uptake in the infarcted myocardium and infarct border zone, we observed several remarkable and so far inexplicable features of our probe. We found the overall uptake to be higher in every section of the infarcted hearts compared with that in the healthy control hearts. This may be due to enhanced post-infarct overall stress in the heart, but we cannot confirm such a finding at this moment. Moreover, kidney uptake was significantly higher in the healthy control mice compared to that in the MI mice. Although we currently have no direct link between the infarct and these findings, these discoveries we made using SPECT scans were overlooked in earlier studies employing two-photon microscopy and MRI and may be of considerable importance for future studies employing the cNGR molecule as a molecular angiogenesis imaging probe.

In this study, we opted for ^111^In due to the fact that pre-clinical SPECT scanners outclass pre-clinical PET scanner in terms of spatial resolution. Of course, the use of ^111^In in a clinical situation may be less favourable due to its long physical half-life of 2.8 days and resolution limitations. While pre-clinical SPECT scanners outclass pre-clinical PET scanners in terms of spatial resolution, in the clinical setting, this is reversed. Therefore, angiogenesis imaging via the cNGR tripeptide in MI patients would benefit from the use of a clinical PET system. Future research should therefore focus on the development of a high-resolution cNGR-based PET imaging probe, for example by replacing ^111^In by the PET isotope ^68^Ga.

## Conclusions

In conclusion, in this study, we designed and developed a molecular angiogenesis imaging probe that allowed us to sensitively visualise CD13 in the border zone of infarcted myocardium and infarcted myocardium itself using dual-isotope SPECT imaging. Analysis of acquired SPECT images in the AHA 17 segment model revealed target-specific uptake of our new angiogenesis imaging probe in areas of decreased perfusion. Furthermore, we noticed rapid clearance via the urinary tract and low uptake in non-specific organs. However, for the cNGR tripeptide to be considered as a clinical angiogenesis imaging tool, specific target uptake has to increase. Therefore, we are currently pursuing a multi-valent cNGR motif with supposed longer half-life and higher target affinity.

## Additional file

Additional file 1: Figure S1. The SUVs of ^111^In-DTPA-cNGR in *ex vivo* scanned Matrigels. The mean SUV of explanted hFGF2-supplemented Matrigels was higher compared to that of the controls (8.238 × 10^−4^ ± 5.823 × 10^−4^ vs. 2.2235 × 10^−4^ ± 7.001 × 10^−5^, *p* = 0.1527 and ratio 3.7 supplemented vs. non-supplemented).

## Abbreviations

cNGR: cyclic asparagine-glycine-arginine
RGD: arginine-glycine-aspartic-acid
DTPA: diethylene triamine pentaacetic acid
LAD: left anterior descending coronary artery
hFGF2: human fibroblast growth factor 2
CF: conversion factor
SUV: standardised uptake value
