# Supplementary figures and images for: Molecular imaging of angiogenesis after myocardial infarction by 111In-DTPA-cNGR and 99mTc-sestamibi dual-isotope myocardial SPECT
Source: EJNMMI Res. 2015 Jan 28;5:2. doi: 10.1186/s13550-015-0081-7 (PMC4384708; doi:10.1186/s13550-015-0081-7)

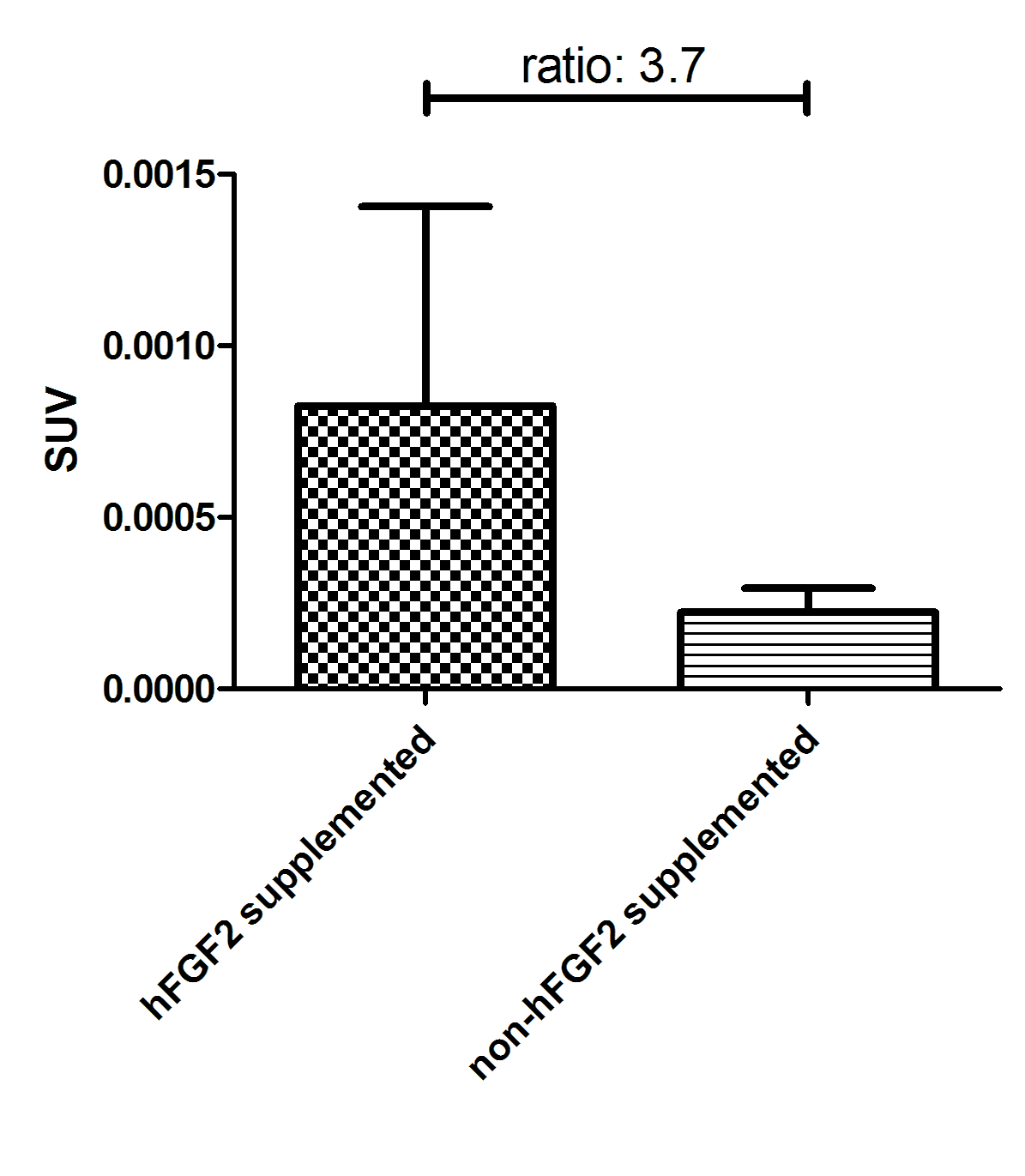

Supplement: Additional file 1: Figure S1. — The SUVs of 111In-DTPA-cNGR in ex vivo scanned Matrigels. The mean SUV of explanted hFGF2-supplemented Matrigels was higher compared to that of the controls (8.238 × 10^−4^ ± 5.823 × 10^−4^ vs. 2.2235 × 10^−4^ ± 7.001 × 10^−5^, p = 0.1527 and ratio 3.7 supplemented vs. non-supplemented). [file 13550_2015_81_MOESM1_ESM.tiff]
